# Identification of DPP4/CTNNB1/MET as a Theranostic Signature of Thyroid Cancer and Evaluation of the Therapeutic Potential of Sitagliptin

**DOI:** 10.3390/biology11020324

**Published:** 2022-02-17

**Authors:** Sheng-Yao Cheng, Alexander T. H. Wu, Gaber El-Saber Batiha, Ching-Liang Ho, Jih-Chin Lee, Halimat Yusuf Lukman, Mohammed Alorabi, Abdullah N. AlRasheedi, Jia-Hong Chen

**Affiliations:** 1 Department of Otolaryngology-Head and Neck Surgery, Tri-Service General Hospital, National Defense Medical Center, 325, Section 2, Chenggong Road, Taipei 114, Taiwan; gjcheng5032@gmail.com (S.-Y.C.); doc30450@gmail.com (J.-C.L.); 2 TMU Research Center of Cancer Translational Medicine, Taipei Medical University, Taipei 110, Taiwan; chaw1211@tmu.edu.tw; 3 The PhD Program of Translational Medicine, College of Science and Technology, Taipei Medical University, Taipei 110, Taiwan; 4 Clinical Research Center, Taipei Medical University Hospital, Taipei Medical University, Taipei 110, Taiwan; 5 Graduate Institute of Medical Sciences, National Defense Medical Center, Taipei 110, Taiwan; 6 Department of Pharmacology and Therapeutics, Faculty of Veterinary Medicine, Damanhour University, Damanhour 22511, Egypt; gaberbatiha@gmail.com; 7 Division of Hematology/Oncology, Department of Medicine, Tri-Service General Hospital, National Defense Medical Center, Taipei 11490, Taiwan; charileho22623@gmail.com; 8 Department of Chemical Sciences, Biochemistry Unit, College of Natural and Applied Sciences, Summit University Offa, Offa PMB 4412, Nigeria; halimatyusuf40@summituniversity.edu.ng; 9 Department of Biotechnology, College of Sciences, Taif University, Taif P.O. Box 11099, Saudi Arabia; maorabi@tu.edu.sa; 10 Otolaryngology-Head & Neck Surgery Department, College of Medicine, Jouf University, Sakaka P.O. Box 2014, Saudi Arabia; analrashedi@ju.edu.sa

**Keywords:** sitagliptin, thyroid cancer (THCA), papillary thyroid cancer (PTCa), thyroidectomy, metastasis, drug resistance

## Abstract

**Simple Summary:**

In recent years, the incidence of thyroid cancer has been increasing globally, with papillary thyroid cancer (PTCa) being the most prevalent pathological type. Although PTCa has been regarded to be slow growing and has a good prognosis, in some cases, PTCa can be aggressive and progress despite surgery and radioactive iodine treatment. Therefore, searching for new targets and therapies is required. We utilized bioinformatics analyses to identify critical theranostic markers for PTCa. We found that DPP4/CTNNB1/MET is an oncogenic signature that is overexpressed in PTCa and associated with disease progression, distant metastasis, treatment resistance, immuno-evasive phenotypes, and poor clinical outcomes. Interestingly, our in silico molecular docking results revealed that sitagliptin, an antidiabetic drug, has strong affinities and potential for targeting DPP4/CTNNB1/MET signatures, even higher than standard inhibitors of these genes. Collectively, our findings suggest that sitagliptin could be repurposed for treating PTCa.

**Abstract:**

In recent years, the incidence of thyroid cancer has been increasing globally, with papillary thyroid cancer (PTCa) being the most prevalent pathological type, accounting for approximately 80% of all cases. Although PTCa has been regarded to be slow growing and has a good prognosis, in some cases, PTCa can be aggressive and progress despite surgery and radioactive iodine treatment. In addition, most cancer treatment drugs have been shown to be cytotoxic and nonspecific to cancer cells, as they also affect normal cells and consequently cause harm to the body. Therefore, searching for new targets and therapies is required. Herein, we explored a bioinformatics analysis to identify important theranostic markers for THCA. Interestingly, we identified that the *DPP4/CTNNB1/MET* gene signature was overexpressed in PTCa, which, according to our analysis, is associated with immuno-invasive phenotypes, cancer progression, metastasis, resistance, and unfavorable clinical outcomes of thyroid cancer cohorts. Since most cancer drugs were shown to exhibit cytotoxicity and to be nonspecific, herein, we evaluated the anticancer effects of the antidiabetic drug sitagliptin, which was recently shown to possess anticancer activities, and is well tolerated and effective. Interestingly, our in silico molecular docking results exhibited putative binding affinities of sitagliptin with *DPP4/CTNNB1/MET* signatures, even higher than standard inhibitors of these genes. This suggests that sitagliptin is a potential THCA therapeutic, worthy of further investigation both in vitro and in vivo and in clinical settings.

## 1. Introduction

Thyroid cancer (THCA) is the most prevalent malignancy of the endocrine system, and the 9th most common cancer in the world [1,2], accounting for approximately 600,000 newly diagnosed cases annually on a global scale [3], with high rates of morbidity reported in recent years [4]. THCA is divided into various subtypes, including anaplastic thyroid cancer (ATC), papillary thyroid carcinoma (PTCa), and follicular thyroid carcinoma (FTC), with PTCa being the most prevalent, as it accounts for approximately 85% of THCA [5,6]. PTC and FTC are well-differentiated thyroid cancers with an optimal prognosis of about 10 years disease-specific survival [7]. However, the ATC is poorly differentiated with proliferative stem-cell-like properties, resistance to therapies, and accounts for the majority of thyroid-cancer-related deaths [8,9]. The rapid increase in thyroid cancer, particularly PTCa, has been accredited to the availability and sensitive use of ultrasonography and other diagnostic imaging modalities [10,11], which have likely led to a massive detection and diagnosis of a large reservoir of subclinical, indolent lesions of the thyroid [12,13]. Studies have also implicated obesity, hormonal imbalance, metabolic syndromes, and environmental pollutants in the development of PTCa [14].

Patients with PTCa usually show good clinical outcomes compared with other cancers; however, there is also a very high rate of relapse post-treatment, leading to distant metastasis [15,16]. About 11% of patients with PTC present with distant metastases outside the neck and mediastinum [17]. Moreover, long-term survival outcomes for aggressive PTC subgroups exhibit heterogeneous clinical behavior and a wide range of mortality risks, suggesting that treatment should be tailored to specific histologic subtypes [18]. The diagnostic criteria for PTC allow it to demonstrate various histological features and growth patterns; different variants of PTCa are recognized, including classic, microcarcinoma, encapsulated, follicular, diffuse sclerosing, tall cell, columnar cell, cribriform-morular, hobnail, solid, oncocytic, spindle cell, clear cell, and Warthin-like variants [19]. However, among these variants, tall cell, columnar cells, and hobnail variants are of undoubted clinical significance, since they are aggressive variants associated with aggressive clinicopathological features and worse prognosis than for classic and encapsulated PTC [20,21,22].

Surgery, endocrine therapy, and radioiodine therapy are well-known therapy regimens for PTCa, offering a good prognosis; however, the aggressive variants of PTCa progress despite surgery and radioactive iodine treatment [23]. In addition, tumor recurrence in PTCa is associated with therapeutic resistance which increases the death toll in patients [24,25,26]. Unfortunately, an upsurge in the incidence of aggressive PTCs was observed at a rate higher than that seen in well-differentiated PTCs or anaplastic thyroid carcinomas (ATCs) in the past two decades in a study of a large cohort of thyroid cancers [22]; therefore, there is an urgent need to identify novel diagnostic and prognostic molecular biomarkers that could also be used as molecular targets for the development of new drugs or in repurposing existing drugs for the treatment of PTCa.

Increasing evidence shows that dipeptidyl aminopeptidase IV (*DPP IV*) is associated with cancer development and progression [27,28]; DPP4 is an adenosine deaminase complex protein, and was demonstrated to be upregulated in THCA, particularly in PTCa, and is associated with tumor aggression and poor prognoses [29,30,31]. Moreover, high expression of *DPP4* was shown to promote distance metastasis and stemness in esophageal adenocarcinoma and colorectal cancer [32,33]. However, the prognostic role of *DPP4* expression and its role in THCA metastasis remains elusive [7,29,31]. Studies have shown that DPP4 and b-catenin crosstalk to regulate critical cellular processes, including motility and invasion [34]. A study involving lung cancer patients has revealed that the expression levels of β-catenin correlate with DPP4 expression [35] and contributed to tumor metastasis [34,36]. An experimental study has also reported that activating mutation of *Ctnnb1* induced DPP4 overexpression in epidermal keratinocytes of LRIG1^+^ stem cells [37]. Research has illuminated that inhibitors of DPP4 exert their therapeutic effect via modulation of the Wnt/β-catenin signaling pathway [38]. Sitagliptin, an inhibitor of DPP4, has also been reported to provide renal protection via inhibition of the tubulointerstitial Wnt/β-catenin signaling pathway in diabetic nephropathy [39].

Accumulating studies demonstrated a pivotal correlation between distant metastasis in PTCa and *MET* (MET proto-oncogenic receptor tyrosine kinase) [40]. Approximately 70% of PTCas were reported to overexpress the *MET* gene, and it is associated with poor prognoses [41]. In addition, Rossana et al. also demonstrated that higher expression levels of *MET* in PTCa promoted cancer growth and distance metastasis [42,43]. *MET* is a transmembrane tyrosine kinase identified as a high-affinity receptor for hepatocyte growth factor (HGF), and both *MET* and *HGF* were demonstrated to be expressed in PTCa [42], and consequently promote progression and secondary metastasis [44]. Additionally, *MET* was shown to activate β-catenin (*CTNNB1*), an important component of the canonical Wnt pathway [45,46]. *CTNNB1* was recently reported to be mutated in PTCa, and to ultimately promote cancer development and stemness [47,48]. Moreover, upregulated *MET* was also demonstrated to regulate the expression of mitogen-activated protein kinase (*MAPK*), phosphatidylinositol 3-kinase (*PI3K*)*/AKT*, signal transducer and activator of transcription 3 (*STAT3*), and nuclear factor (*NF*)*-κB* pathways in THCA [40,49]. This suggests that *MET* is a crucial target gene in THCA, and worthy of further investigation. To date, most drugs used for cancer treatment are cytotoxic and usually not specific to cancer cells, but also affect normal cells; therefore, there is still a huge gap in finding more sensitive and specific drugs for cancer. Recent studies suggested an association between cancer occurrence and antidiabetic medicaments. Sitagliptin is a standard inhibitor of *DPP4*, widely used for treating diabetes, and was shown to possess anticancer activities, as well as being efficacious and well tolerated [50]. In the present study, we predicted the potential anticancer activities of sitagliptin as a target for *DPP4/CTNNB1/MET* oncogenic signatures, which are overexpressed in THCA.

## 2. Materials and Methods

### 2.1. Microarray Data Acquisition and Identification of Differentially Expressed Genes (DEGs)

Gene expressions of four THCA datasets (GEO3467, GEO36787, GEO6004, and GEO33630) were extracted from the NCBI gene expression omnibus. The acquired datasets were further analyzed using GEO2R (https://www.ncbi.nlm.nih.gov/geo/geo2r/ accessed on 5 September 2021), and results contained DEG profiles from THCA patients compared to normal samples. To control the false discovery rate (FDR), the Benjamini–Hochberg adjustment was applied to *p* values (adjusted (adj.) *p* values), to moderate the balance between detection of significant genes and possible false-positive values. The fold-change (FC) threshold was set to 1.5, and adj. *p* < 0.05 was considered statistically significant. Venn diagrams were constructed using the Bioinformatics and Evolutionary Genomics (BEG) online tool (http://bioinformatics.psb.ugent.be/webtools/Venn/ accessed on 6 September 2021).

### 2.2. Differential Expression of the THCA Gene Hub

Differential expressions of THCA gene profiles between tumor tissues and normal adjacent tissues of the Cancer Genome Atlas (TCGA) database were analyzed using UALCAN (http://ualcan.path.uab.edu accessed on 12 September 2021), an online web portal used to identify gene expression levels between primary tumors compared to normal tissue samples [51]. Moreover, we explored the cBioPortal online web tool (https://www.cbioportal.org accessed on 19 September 2021), which categorizes gene alterations based on percentages of individual genes due to amplification [52]. For further analysis, we used the cBioPortal correlation sub-tool to determine gene expression correlations with positive Spearman and Pearson correlation coefficients with *p* < 0.05 as statistically significant.

### 2.3. Comparisons of DPP4/CTNNB1/MET Expressions in Normal, Primary, and Metastatic Tumor of Thyroid Cancer Cohorts

To compare expression levels of the *DPP4/CTNNB1/MET* oncogenes among normal, tumor, and metastatic tissues, we explored the tumor, normal, and metastatic plot (TNMplot), (https://tnmplot.com/analysis/ accessed on 21 September 2021), an RNA-sequence-based rapid analysis, which is used to compare data of selected genes [53]. Data were compared using the Kruskal–Wallis test, which is a method used to test samples originally from the same distribution of specimens, followed by Dunn’s test, which assesses the significance of gene expressions in promoting THCA tumor metastasis, with *p* < 0.05 considered statistically significant.

### 2.4. Interaction Network and Gene Enrichment Analysis

An interaction network analysis was constructed using the Search Tool for the Retrieval of Interacting Genes/Proteins (STRING, https://string-db.org/ accessed on 25 September 2021) database [54], and GeneMANIA [55] (http://genemania.org/data accessed on 28 September 2021), which are online web tools developed to analyze interaction networks. The STRING database was used under a high confidence of 0.700, and protein enrichment of *p* < 6.0 × 10^−03^ was obtained. Interactions among genes were analyzed according to correlations based on experimental data (pink), gene neighborhoods (green), gene fusion (red), gene co-occurrences (blue), and gene co-expression (black). Moreover, we explored the Network Analyst user-friendly online tool (https://www.networkanalyst.ca/ accessed on 5 October 2021) to analyze co-expressed gene enrichment from the biological processes databases; herein we applied the Igraph R package visualization tool for analysis [56]. Furthermore, gene ontology (GO), biological processes (BPs), and Kyoto Encyclopedia of Genes and Genomes (KEGG) enrichment analyses were analyzed using FunRich software (http://www.funrich.org accessed on 9 October 2021), an open access, stand-alone functional enrichment and network analytical tool [57].

### 2.5. Analysis of Genomic Alterations and Mutations of the DPP4/CTNNB1/MET Oncogenes in THCA

Mutations of *DPP4/CTNNB1/MET* oncogenic expressions in THCA were analyzed using cBioPortal software. Herein, we analyzed altered frequencies of these oncogenes in THCA. Furthermore, we explored the muTarget platform (https://www.mutarget.com/ accessed on 11 October 2021), a platform linking changes in gene expressions and the mutation status of solid tumors, based on a genotype analysis, to determine associations between *DPP4/CTNNB1/MET* and alterations in gene expressions in THCA. Differences in expressions between the mutant group and wild-type (WT) group were considered statistically significant at *p* < 0.05.

### 2.6. Correlations of DPP4/CTNNB1/MET Expressions and Tumor Infiltration Levels of Immune and Immunosuppressive Cells in THCA

The Tumor Immune Estimation Resource (TIMER) (https://cistrome.shinyapps.io/timer/ accessed on 18 October 2021) is an online computational tool used to analyze the nature of tumor immune interactions across different cancer types [58]. Herein, we determined correlations of *DPP4/CTNNB1/MET* expressions and tumor infiltration levels of tumor associated macrophages (M2 TAM), regulatory T cell (Treg), cancer-associated fibroblast (CAF), and cluster of differentiation 8-positive (CD8^+^ T cell), using a set of gene markers of immune infiltration model, as described previously [59,60]. The strength of correlations between the genes and immune cells is reflected by the purity-adjusted partial Spearman’s rho value, where a value of r ≥1 means a perfect positive correlation and a value of r ≤ −1 means a perfect negative correlation, with *p* < 0.05 considered statistically significant.

### 2.7. In Silico Molecular Docking of the DPP4/CTNNB1/MET Oncogenes with Sitagliptin

The potential inhibitory effects of sitagliptin on THCA hub genes of *DPP4*, *CTNNB1*, and *MET* were analyzed by molecular docking simulations, compared to the standard inhibitors of CTNNB1 and MET of PNU-74654 and crizotinib, respectively. The 3D structures of sitagliptin (CID: 4369359), PNU-74654 (CID:9836739), and crizotinib (CID:116250) were retrieved from the pubchem database (https://pubchem.ncbi.nlm.nih.gov/ accessed on 22 October 2021), in the spatial data file (SDF) format, and consequently converted to PDB file format using the PyMOL visualization tool [61] (https://pymol.org/2/ accessed on 22 October 2021), while the crystal structures of DPP4 (PDB:2ONC), CTNNB1 (PDB:1JDH), and MET (PDB:3DKF) were downloaded from the protein database (PDB), (https://www.rcsb.org/ accessed on 22 October 2021), in PDF file format. File preparation for molecular docking was as described in previous studies [62,63,64]. Using autodock software, an in silico molecular docking tool [65], all PDB files were converted to PDBQT file formats, and docking was accordingly performed using autodock, as described previously [66,67]. For further analysis, we used PyMol to analyze ligand–receptor interactions in 3D view, and finally used the discovery studio web tool [68] for data interpretation.

## 3. Results

### 3.1. Identification of Common Oncogenes in THCA

**Figure 1:**
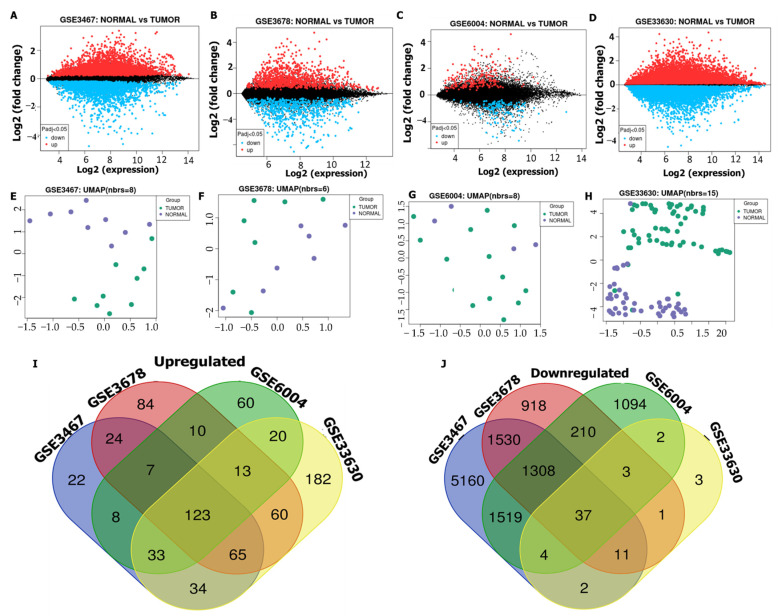
Differentially expressed genes (DEGs) in thyroid cancer (THCA). (**A**–**D**) Volcano plots showing DEGs extracted from the GSE3467, GSE3678, GSE6004, and GSE33630 microarray datasets, between cancer tissues compared with normal adjacent tissues, with upregulated genes (red), downregulated genes (blue), and non-significant genes (black). (**E**–**H**) Two-dimensional (2D) visualization of UMAP dimensionality reduction in THCA tumor tissues (green) compared with normal tissues (purple). (**I**,**J**) Venn diagram of 123 overlapping DEGs between normal colon tissues and tumor tissues.

Microarray datasets were downloaded from the NCBI-GEO database to identify DEGs in THCA. Commonly expressed oncogenes were identified from THCA tissues compared to adjacent normal tissues obtained from different studies. Volcano plots were used to show all DEGs from all selected datasets, and accordingly, the GSE3467, GSE3678, GSE6004, and GSE33630 datasets, respectively, displayed 691, 449, 1455, and 789 upregulated genes and 1088, 1232, 2890, and 1568 downregulated genes (Figure 1A–D). The relatedness of all samples in each dataset to each other was analyzed by uniform manifold approximation and projection (UMAP), in which the number of nearest neighbors was used for calculations as indicated in each plot (Figure 1E–H). In total, 123 overlapping genes were obtained using Venn diagrams, as observed from THCA tissues compared with normal tissues (Figure 1I,J). We further used these genes for further analysis of THCA in this study.

### 3.2. DPP4/CTNNB1/MET Expressions Are Associated with THCA Progression, Metastasis, and Worse Prognosis of THCA Cohorts

**Figure 2:**
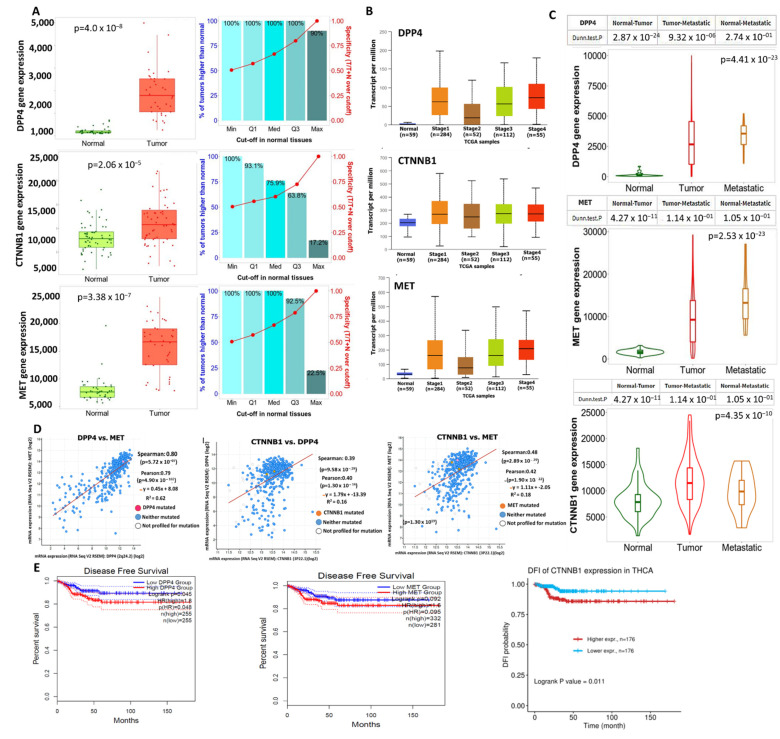
Overexpression of *DPP4/CTNNB1/MET* mRNAs, associated with thyroid cancer (THCA) progression. Differential expression levels of *DPP4/CTNNB1/MET* between (**A**) THCA tumor and adjacent normal tissue, (**B**) tumor stages, and (**C**) between primary and metastatic tumor of TCGA cohort. (**D**) Correlations of *DPP4* with *MET, CTNNB1* with *DPP4*, and *MET* with *CTNNB1* oncogenic expressions in THCA. (**E**) KPM plots of survival ratio between THCA cohorts with high and those with low expression levels of *DPP4/CTNNB1/MET*.

Our differential expression analysis revealed that the (m)RNA expression levels of *DPP4/CTNNB1/MET* were higher in THCA tumor tissues compared with adjacent normal tissues (Figure 2A). We further analyzed the role of *DPP4*, *CTNNB1*, and *MET* expressions in promoting THCA progression and tumor metastasis. Interestingly, our analysis revealed that the mRNA expressions levels of *DPP4/CTNNB1/MET* were more elevated in stage IV of THCA cancer (Figure 2B), and were significantly elevated in metastasis tumor compared with the primary tumor (Figure 2C). In addition, we found expression correlation among the *DPP4/CTNNB1/MET* signature in THCA cohorts (Figure 2D). Furthermore, we constructed a Kaplan–Meier (KM) plot of patients’ survival and found that higher expression levels of the *DPP4/CTNNB1/MET* genes were associated with shorter survival duration of the cohorts (Figure 2E). Although the KM plot revealed no significant (*p* > 0.05) difference in the overall survival between cohorts with high and cohorts with low expression levels of DPP4, our analysis revealed that the disease-free survival of the cohorts was significantly (*p* < 0.048) higher in the low-DPP4-expression group when compared with the high-expression group. Collectively, our findings strongly suggested that the expression levels of DPP4/CTNNB1/MET signature are associated with THCA progression, metastasis, and worse prognosis of THCA cohorts, hence serving as important biomarker for diagnosis, prognosis, and therapeutic exploration in THCA.

### 3.3. DPP4/CTNNB1/MET Genes Are Frequently Altered and Their Mutations Are Linked to Genetic Expressions in THCA

**Figure 3:**
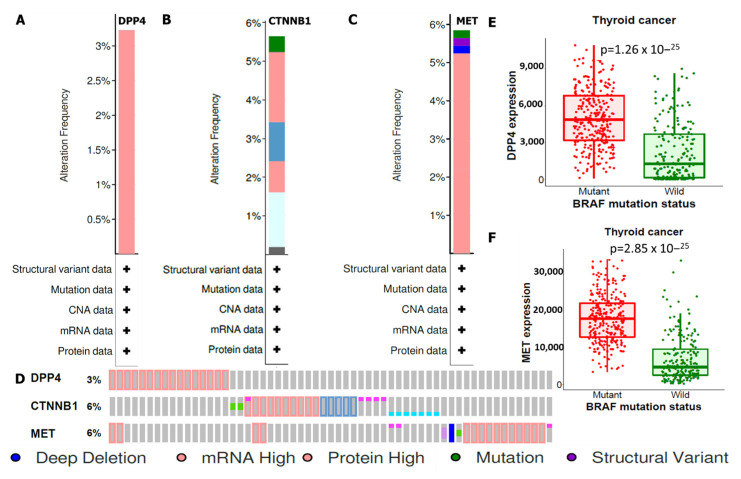
Genetic mutations based on percentages due to amplification of (**A**) *DPP4* (3%), (**B**) *CTNNB1* (6%), and (**C**) *MET* (6%), including deep deletions (blue), mRNAs (red), proteins (red), mutations (green), and structural variants (purple). (**D**) Individual genetic alteration profile of DPP4/CTNNB1/MET in THCA. (**E**,**F**) *BRAF* mutations promoted overexpression of *DPP4* and *MET* compared with the wild type, with *p* < 0.05 considered statistically significant.

Mutations of *DPP4/CTNNB1/MET* oncogenes in THCA were analyzed using the cBioPortal tool, and altered frequencies were based on percentages of individual genes due to amplification. Analytical results showed respective amplification of *DPP4*, *CTNNB1*, and *MET* occur in 3%, 6%, and 6% of THCA cohorts respectively. These included deep deletions (blue), mRNAs (red), proteins (red), mutations (green), and structural variants (purple) (Figure 3A–D). For further analysis, we compared associations between alterations in *DPP4* and *MET* oncogenic expressions with mutations of the top genes expressed in THCA at the target level, and according to our findings, *BRAF* mutations promoted increased expression levels of *DPP4* and MET compared with the WT. Patients with high expression levels of *DPP4* and *MET* signatures exhibited worse clinical outcomes compared with patients with low expression levels (Figure 3E,F).

### 3.4. DPP4/CTNNB1/MET Genes Potentially Promote Tumor Growth by Interacting with Different Oncogenic Targets/Pathways

**Figure 4:**
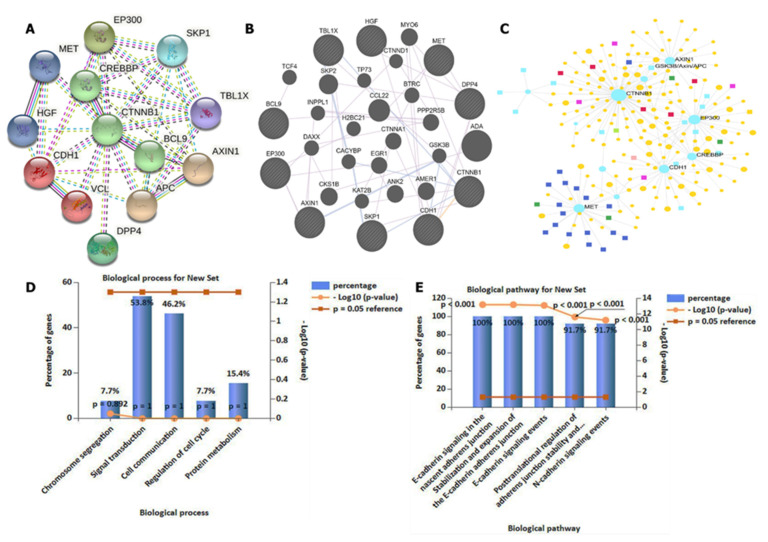
*DPP4/CTNNB1/MET* gene interactions co-expressed in the same clustering network. (**A**,**B**) Interaction networks showing co-expression between *DPP4* and *CTNNB1*, *MET* and *DPP4*, *CTNNB1* and *MET*, *HFG* and *MET*, *DPP4* and *CTNND1*, and *GSK3B* and *CTNND1* within the network clustering. An average local clustering coefficient of 0.787 was obtained, with an expected number of edges of 21 and an interaction *p* value of 0.006. (**C**) Gene enrichment analysis gene ontology (GO) showed enrichment in co-expressions of *CTNNB1*, *GSK3B*, *AXIN1*, and *MET* in biological processes. (**D**,**E**) Validation of GO, involving enrichment of the top five pathways involved, with *p* < 0.05 considered significant.

We applied the STRING database and GeneMANIA online web tools developed to analyze interaction networks among four selected oncogenes. Herein, we considered experimental data (pink), gene neighborhoods (green), gene fusion (red), gene co-occurrences (blue), and gene co-expressions (black) when analyzing interactions. As expected, interaction networks were identified between *DPP4* and *CTNNB1*, *MET* and *DPP4*, *CTNNB1* and *MET*, *HFG* and *MET*, *DPP4* and *CTNND1*, and *GSK3B* and *CTNND1* within the network clustering. An average local clustering coefficient of 0.787 was obtained, with an expected number of edges of 21 and interaction *p* value of 0.006 (Figure 4A,B). For further analysis, we conducted a gene enrichment analysis and predicted GO processes using network analytical software, which showed co-expressions of *CTNNB1*, *GSK3B*, *AXIN1*, and *MET* to be enriched in the BP databases. Herein, we applied the Igraph R package visualization tool for analysis (Figure 4C). For more analysis, we used FunRich software to validate GO including BPs and KEGG enrichment analyses. The top five enriched BPs included chromosomal segregation, signaling transduction, cell communication, regulation of the cell cycle, and protein metabolism, while pathways involved in interactions included E-cadherin signaling in the nascent cadherin junction, stabilization and expression of adherens junctions, E-cadherin signaling events, posttranscriptional regulation of adherens junction stability, and N-cadherin signaling events (Figure 4D,E), with *p* < 0.05 considered significant.

### 3.5. High Expression Levels of DPP4/CTNNB1/MET Are Associated with Immunosuppressive Phenotypes of THCA Tissues

**Figure 5:**
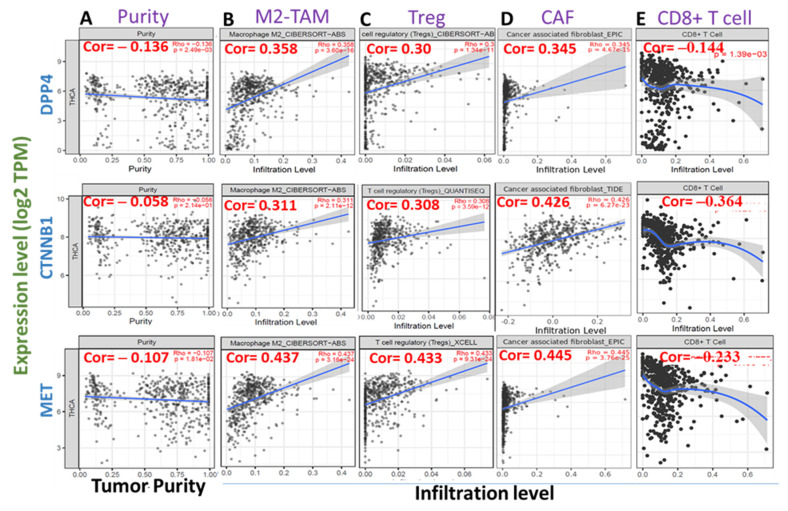
High expression levels of DPP4/CTNNB1/MET are associated with immunosuppressive phenotypes of THCA tissues. Scatterplots of *DPP4/CTNNB1/MET* expression correlations with (**A**) tumor purity, and infiltration levels of (**B**) tumor-associated macrophages (M2 TAM), (**C**) regulatory T cell (Treg), (**D**) cancer-associated fibroblast (CAF), and (**E**) CD8^+^ T cell. The strength of correlations between the genes and immune cells is reflected by the purity-adjusted partial Spearman’s rho value, where a value of r ≥1 means a perfect positive correlation and a value of r ≤ −1 means a perfect negative correlation, with *p* < 0.05 considered statistically significant.

We queried the association between the mRNA expression levels of DPP4/CTNNB1/MET and tumor infiltrations of immunosuppressive cells using the TCGA cohorts. Interestingly, we found that the mRNA expression levels of DPP4/CTNNB1/MET are inversely associated with tumor purity (Figure 5A). In addition, the high expression levels of the DPP4/CTNNB1/MET correlate positively (all *p* < 0.001, cor > 0.3) with the infiltration levels of tumor-associated macrophages (M2 TAM Figure 5B), regulatory T cell (Treg, Figure 5C), and cancer-associated fibroblast (CAF, Figure 5D) in thyroid cancer cohorts (Figure 5). In contrast, a strong negative association (all *p* < 0.001, cor < 0) was observed between the mRNA expression levels of *DPP4/CTNNB1/MET* and the immune infiltration level of CD8^+^ T cell (Figure 5E), an antitumor T cell subtype. Collectively, these findings strongly suggested that high expression levels of DPP4/CTNNB1/MET are associated with immunosuppressive phenotypes via a mechanism involving T cell exclusion in THCA tissues.

### 3.6. Molecular Docking Reveals Higher Inhibitory Effects of Sitagliptin on the DPP4 Oncogene

**Figure 6:**
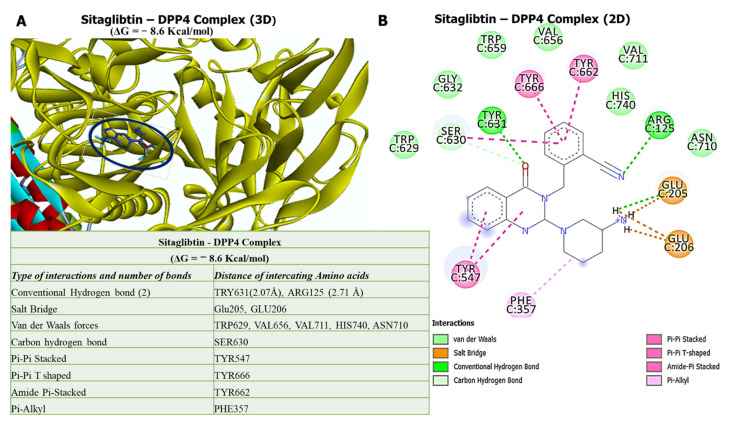
Ligand–receptor interaction results of sitagliptin with *DPP4.* (**A**) Three-dimensional (3D) representation of sitagliptin in complex with *DPP4* with the highest binding energy of −8.6 kcal/mol. (**B**) Two-dimensional (2D) representation of sitagliptin in complex with *DPP4*, showing interactions with two conventional H-bonds, with interactions further stabilized by different amino acids around the sitagliptin backbone. The accompanying table shows summary results of the analysis.

Our in silico molecular docking analysis revealed that sitagliptin exhibited higher binding energy of −8.6 kcal/mol with the *DPP4* oncogene. Further analysis of the docking results showed that sitagliptin bound to the binding pocket of the *DPP4* gene by hydrogen bonds with shorter binding distances at TRY631 (2.07 Å) and ARG125 (2.71 Å), and was further stabilized by a salt bridge, van der Waals forces, carbon–hydrogen bonds, Pi-Pi stacked, Pi-Pi T-shaped, amide Pi-stacked, and Pi-alkyl around the sitagliptin backbone (Figure 6).

### 3.7. Molecular Docking Revealed Potential Inhibitory Effects of Sitagliptin on the CTNNB1 Oncogene

**Figure 7:**
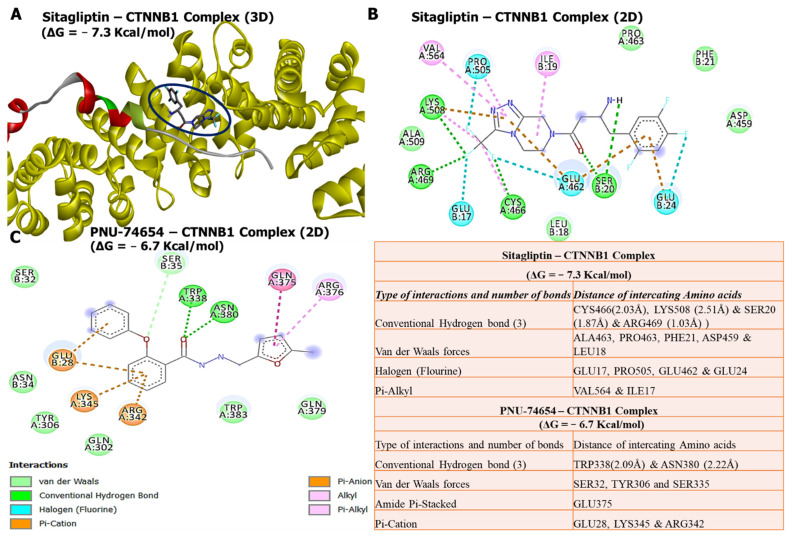
In silico molecular docking analysis of ligand–protein interactions. (**A**) Three-dimensional (3D) representation of sitagliptin in complex with *CTNNB1* with a binding energy of −7.3 kcal/mol. (**B**) Two-dimensional (2D) representation of sitagliptin in complex with *CTNNB1*, showing interactions with four conventional H-bonds and shorter binding distances, with interactions further stabilized by different amino acids around the sitagliptin backbone. (**C**) Two-dimensional (2D) representation of PNU-74654 in complex with *CTNNB1*, displaying lower binding energy of −6.7 kcal/mol, and interactions with (2) conventional hydrogen bonds with longer binding distances compared with that of sitagliptin, in the binding pockets of *CTNNB1*. The accompanying table shows a summary of the results.

Our docking analysis revealed that sitagliptin exhibited high binding energy of −7.3 kcal/mol with the *CTNNB1* oncogene, compared with its Food and Drug Administration (FDA)-approved inhibitor, PNU-74654, which showed a lower binding affinity of −6.7 kcal/mol. Further analysis of the docking results showed that sitagliptin bound to the binding pocket of the *CTNNB1* oncogene by 4 conventional hydrogen bonds and shorter binding distances with CYS466 (2.03 Å), LYS508 (2.51 Å), SER20 (1.87 Å), and ARG469 (1.03 Å). The interactions were further stabilized by van der Waals forces with ALA463, PRO463, PHE21, ASP459, and LEU18, halogen (fluorine) with GLU17, PRO505, GLU462, and GLU24, and Pi-alkyl with VAL564 and ILE17 around the sitagliptin backbone. The results were further compared with the PNU-74654/*CTNNB1* complex, which is bound to the binding pocket of the *CTNNB1* oncogene by only two conventional hydrogen bonds and longer binding distances compared with the sitagliptin/*CTNNB1* complex. The interactions were further stabilized by van der Waals forces with SER32, TYR306, and SER335, amide Pi-stacked with GLU375, and Pi-cation with GLU28, LYS345, and ARG342 around the PNU-74654 backbone. This suggests that sitagliptin has a high potential to target β-catenin (*CTNNB1*), compared with its standard inhibitor, PNU-74654 (Figure 7).

### 3.8. Molecular Docking Revealed Potential Inhibitory Effects of Sitagliptin on the MET Oncogene

**Figure 8:**
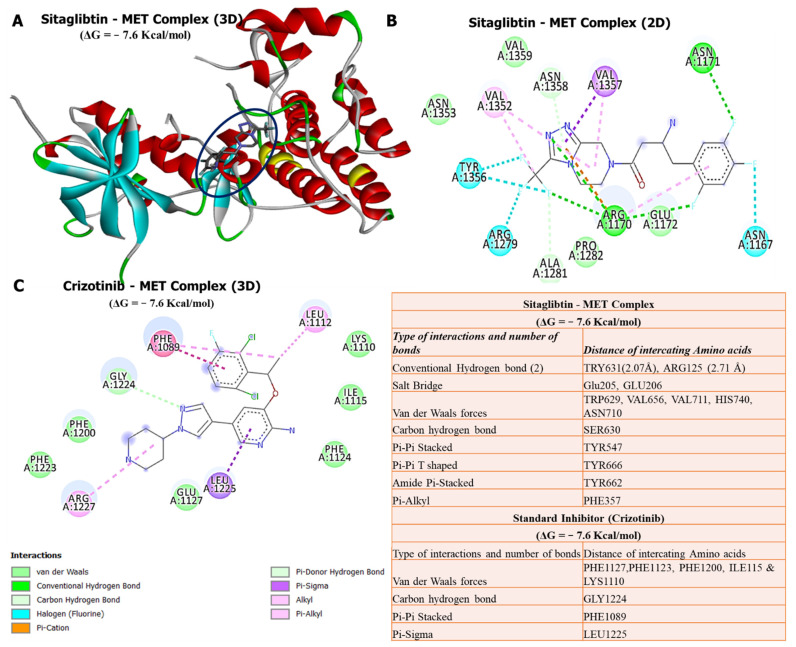
In silico molecular docking analysis of ligand–protein interactions. (**A**) Three-dimensional (3D) representation of sitagliptin in complex with *MET* with a binding energy of −7.6 kcal/mol. (**B**) Two-dimensional (2D) representation of sitagliptin in complex with *MET*, showing interactions with conventional H-bonds and different amino acids. (**C**) Two-dimensional (2D) representation of crizotinib in complex with *MET*, exhibiting the same binding energy as sitagliptin, but no interaction with conventional hydrogen bonds. The accompanying table shows a summary of the results.

Our docking analysis revealed that sitagliptin exhibited a high binding energy of −7.6 kcal/mol with the *MET* oncogene, the same as its FDA-approved inhibitor, crizotinib, which showed a binding affinity of −7.6 kcal/mol. Further analysis of the docking results showed that sitagliptin bound to the binding pocket of the *MET* oncogene by 4 conventional hydrogen bonds with shorter binding distances with TRY631 (2.07 Å) and ARG125 (2.71 Å). Interactions were further stabilized by a salt bridge (GLU205 and GLU206), van der Waals forces (TRP629, VAL656, VAL711, HIS740, and ASN710), carbon–hydrogen bond (SER630), Pi-Pi stacked (TYR547), Pi-Pi T-shaped (TYR666), amide Pi-stacked (TYR662), and Pi-alkyl (PHE357) around the sitagliptin backbone. However, results displayed of the crizotinib/*MET* complex did not exhibit conventional hydrogen bonds in the binding pocket of the *MET* oncogene. This suggests that sitagliptin has high potential to target *MET*, compared with its standard inhibitor, crizotinib (Figure 8).

## 4. Discussion

PTCa is the most prevalent type of THCA, which accounts for approximately 80% of all THCAs, consequently promoting cancer invasion, metastasis, and mortality in patients [69,70]. PTCa has recently been managed with a thyroidectomy; however, due to distant metastasis, THCA tends to be extremely aggressive, and resistant to treatment leading to poor prognoses [71,72,73]. Treatment modalities for THCA include the use of doxorubicin, but this has proven not to be very effective due to the development of resistance [1,74,75,76]. As a result, there is an urgent need to understand the molecular mechanisms associated with THCA metastasis, which will help in developing more effective treatments [15,77]. Identification of reliable biomarkers which can be used as diagnostic measures is urgently needed in PTCa. Most cancer therapeutic drugs have been shown to be cytotoxic and nonspecific to cancer cells, as they also affect normal cells and consequently cause harm to the body.

In the present study, we evaluated the anticancer effects of the antidiabetic drug sitagliptin, which was recently shown to possess anticancer activities, and is well tolerated and effective. Sitagliptin is an FDA-approved *DPP4* oncogene [78]. To further analyze sitagliptin, we explored computer-based simulations to identify and predict target genes, which are commonly overexpressed and associated with THCA invasion, progression, metastasis, poor prognosis, and resistance to therapeutics. We utilized microarray datasets from the NCBI-GEO, and identified DEGs in THCA compared to normal tissues. Among the top upregulated genes, were the *DPP4*, *CTNNB1*, and *MET* oncogenes. To validate their expressions, we used the UALCAN online bioinformatics tool with default settings, which showed that mRNA levels of *DPP4/CTNNB1/MET* were higher in THCA tumor tissues compared with adjacent normal tissues. Moreover, after exploring the TNMplot software, for further analysis, we identified that overexpression of *DPP4/CTNNB1/MET* gene signatures promoted THCA metastasis, and were associated with poor disease-free survival and poor prognoses.

The complex and dynamic interactions of immune cells, stoma, and cancer cells within the tumor microenvironment (TME) play a pivotal role in tumor invasion, cancer progression, and host immune response [62,79]. Consequently, our analysis of tumor immune infiltrating cells within the TME of THCA tumor revealed that the high expression levels of the DPP4/CTNNB1/MET signature correlate positively with the infiltration levels of tumor-associated macrophages, regulatory T cell, and cancer-associated fibroblast. These immunosuppressive cells are known to exert an inhibitory role on cytotoxic lymphocytes’ function leading to T cell exclusion and tumor invasive phenotype [59,80]. In contrast, we found a strong negative association was observed between the mRNA expression levels of DPP4/CTNNB1/MET and immune infiltration level of CD8^+^ T cell, suggesting that high expression levels of DPP4/CTNNB1/MET are associated with immunosuppressive phenotypes via a mechanism involving T cell exclusion in THCA tissues

Molecular docking has become an increasingly important tool commonly used to understand drug bimolecular interactions with the target proteins for rational drug design and development [62,81,82]. It is useful in estimating binding affinities of the ligand to the proteins and in providing preliminary mechanistic insight into the behavior of a small molecule drug in the binding cavity of target proteins [83,84], as well as elucidating the potential drug-regulated biochemical processes [79,85]. Consequently, we conducted a molecular docking analysis of interactions of *DPP4/CTNNB1/MET* gene signatures with sitagliptin. As expected, sitagliptin exhibited a higher binding energy of −8.6 kcal/mol with the *DPP4* oncogene. Furthermore, our docking analysis revealed that sitagliptin exhibited a higher binding energy of −7.3 kcal/mol with the *CTNNB1* oncogene compared with its FDA-approved inhibitor, PNU-74654, which showed a lower binding affinity of −6.7 kcal/mol. Our analysis showed that sitagliptin bound to the binding pocket of the *CTNNB1* oncogene by 4 conventional hydrogen bonds and had shorter binding distances with CYS466 (2.03 Å), LYS508 (2.51 Å), SER20 (1.87 Å), and ARG469 (1.03 Å) compared with PNU-74654, which bound to the binding pocket of the *CTNNB1* oncogene by only 2 conventional hydrogen bonds, and had longer binding distances compared with sitagliptin. In addition, analytical results of sitagliptin in complex with *MET* exhibited the same binding energy of −7.6 kcal/mol as the *MET* FDA-approved inhibitor, crizotinib. Sitagliptin bound to the binding pocket of the *MET* oncogene by 4 conventional hydrogen bonds and shorter binding distances with TRY631 (2.07 Å) and ARG125 (2.71 Å). However, results displayed from the crizotinib/*MET* complex did not exhibit conventional hydrogen bonds in the binding pocket of the *MET* oncogene.

In summary, these docking results suggest that sitagliptin has high potential to target *DPP4/CTNNB1/MET* signaling pathways in THCA compared with their standard inhibitors. Since recent studies have shown the efficacy and tolerance of sitagliptin as cancer therapeutic, it would be interesting to further investigate its activities as a target for *DPP4/CTNNB1/MET* signaling pathways in THCA, both in vitro and in vitro in tumor-bearing mice.

## 5. Conclusions

In summary, we revealed that *DPP4*, *CTNNB1*, and *MET* oncogenic signatures are overexpressed in THCA, and are associated with cancer progression, metastasis, resistance, poor disease-free survival, and unfavorable clinical outcomes. Moreover, an in silico molecular docking study exhibited putative binding affinities of sitagliptin with the abovementioned oncogenes, which were higher than the standard inhibitors of these genes. This suggests that sitagliptin could be a potential THCA therapeutic, since it has been shown to be more tolerable and effective in different cancers.

## Data Availability

The datasets generated and analyzed in this study can be made available upon reasonable request.
